# Serum GM3(d18:1-16:0) and GM3(d18:1-24:1) levels may be associated with lymphoma: An exploratory study with haematological diseases

**DOI:** 10.1038/s41598-019-42817-3

**Published:** 2019-04-19

**Authors:** Masako Nishikawa, Makoto Kurano, Takahiro Nitta, Hirotaka Kanoh, Jin-ichi Inokuchi, Yutaka Yatomi

**Affiliations:** 1 Department of Clinical Laboratory Medicine, Graduate School of Medicine, The University of Tokyo, Tokyo, 113-8655 Japan; 2 Division of Glycopathology, Institute of Molecular Biomembrane and Glycobiology, Tohoku Medical and Pharmaceutical University, Sendai, Miyagi 981-8558 Japan

**Keywords:** Glycolipids, Mass spectrometry

## Abstract

GM3 (monosialodihexosylganglioside) is a type of ganglioside, which is a molecule composed of ceramide and oligosaccharide containing one or more sialic acids. Since GM3 is abundantly expressed in blood cells, we investigated the association between GM3 molecular species and haematological diseases. We measured the serum levels of seven GM3 molecular species in subjects with various haematological diseases (n = 52) and healthy subjects (n = 24) using a liquid chromatography tandem-mass spectrometry technique as an exploratory study. In all the subjects with haematological diseases, GM3(d18:1-16:0) were inversely correlated with the erythrocytes counts. Regarding the difference in serum GM3 molecular species levels among each haematological diseases and healthy subjects, the levels of GM3(d18:1-16:0) and GM3(d18:1-24:1) were higher in the lymphoid neoplasm group than healthy subjects. Principal component analyses also revealed that the GM3(d18:1-16:0) and GM3(d18:1-24:1) levels were significant contributing factors for discriminating the lymphoid neoplasm group. Moreover, in the lymphoid neoplasm group, the GM3(d18:1-16:0) levels were significantly and positively correlated with the levels of C-reactive protein, soluble interleukin-2 receptor, and lactate dehydrogenase. In conclusion, in our exploratory study with haematological diseases, GM3 molecular species showed different distribution among disease groups, and serum GM3(d18:1-16:0) and GM3(d18:1-24:1) might be associated with lymphoma.

## Introduction

Gangliosides are a subgroup of glycosphingolipids (GSLs) that consist of a hydrophobic ceramide component and a hydrophilic oligosaccharide component with one or more sialic acid residues. GSLs are well-known as ABO blood group antigens and are mainly located outside the cell membrane, where they form caveolae microdomains (lipid rafts) together with sphingomyelin and cholesterol. Thus, the structural features of GSLs affect the interactions between cells and receptor mediated signal transduction by modulating membrane fluidity and microdomain formation^1^. Actually, gangliosides are reportedly involved in the regulation of numerous biological events at the cellular level, including cellular proliferation^2^, differentiation^3–5^, intracellular signal pathways, and interactions between cells^6,7^. In concordance with these potential biological properties, gangliosides have been reported to be associated with various diseases such as lysosomal storage disorders, Alzheimer’s disease, hearing impairment, and metabolic disorders^8,9^.

Among gangliosides, GM3 (monosialodihexosylganglioside) is most widely distributed ganglioside in the human body, it has the simplest structure, contains one glucose, one galactose, and one sialic acid. GM3 is a metabolic precursor for the formation of more complex gangliosides. GM3 is reportedly abundant in liver and adipose tissue, wherein GM3 accounts for the percentage of 80~90% of total ganglioside content^10^, while in serum, the percentage of GM3 is about 48% of total ganglioside content^11^.

Ganglioside derives from ceramide in the following manner; ceramide is glucosylated to glucosylceramide by glucosylceramide synthase, then glucosylceramide is converted to lactosylceramide. Finally, GM3 is synthesized from lactosylceramide and sialic acid by GM3 synthase. The structural diversity of gangliosides arises from the ceramide component and the oligosaccharide component, resulting in the existence of hundreds of molecular species. Ceramide acyl chains vary in the length of their carbon backbones, degree of saturation, and the presence/absence of α-hydroxylation^12^, and the type of oligosaccharide determines the ganglioside molecular species.

Regarding the association between serum GM3 levels and human diseases, the serum GM3 concentration is reportedly higher in patients with type 2 diabetes, hyperlipidemia, or obesity^9^, and the serum levels of GM3, especially GM3(d18:1-h24:1), are strongly correlated with several risk factors for metabolic diseases^13^.

Contrary to the emerging significance of GM3 levels in metabolic diseases, the association between haematological diseases and GM3 molecular species remains to be investigated in human subjects, although a series of elegant studies have demonstrated an association between GM3 and blood cells; GM3 was first identified in horse erythrocyte membrane^14^ and is also abundant in monocytes and platelets^15^. Regarding the biological effects of GM3 on blood cells, GM3 has been shown to play crucial roles in the induction of differentiation in several blood cell lines^5^ and to determine the direction of differentiation in pluripotent K562 cells^3^. Actually, in ganglioside synthase-deficient mice, the pattern of ganglioside species affected the differentiation of the lymphocyte subsets^16^.

Considering this background, we measured the levels of GM3 species in samples obtained from subjects with haematological diseases and healthy subjects using a liquid chromatography tandem-mass spectrometry (LC-MS/MS) technique to investigate the association between GM3 molecular species and haematological diseases as an exploratory study.

## Results

### Determinants of serum GM3 levels differed among GM3 molecular species

First, we measured the serum concentrations of the GM3 molecular species in subjects with haematological diseases and healthy subjects. Table 1 shows the clinical diagnoses based on the pathological diagnostic criteria of the World Health Organization classification^17^ in subjects with haematological diseases and baseline factors. We observed no significant difference in age and gender ratio among healthy subjects and the subjects with haematological disorders. The total GM3 concentrations were similar between these two groups (4.66 ± 1.65 ng/μL for subjects with haematological diseases vs. 4.28 ± 0.656 ng/μL for healthy subjects). However, we observed that several GM3 species were significantly modulated in the subjects with haematological disorders. For example, the serum GM3(d18:1-16:0) and GM3(d18:1-24:1) levels were significantly higher (GM3[d18:1-16:0]: 1.44 ± 0.557 ng/μL for subjects with haematological diseases vs. 1.10 ± 0.222 ng/μL for healthy subjects [*P* = 0.005]; GM3[d18:1-24:1]: 1.14 ± 0.470 ng/μL for subjects with haematological diseases vs. 0.818 ± 0.159 ng/μL for healthy subjects [*P* < 0.001]) and the serum GM3(d18:1-18:0) level was significantly lower (0.509 ± 0.195 ng/μL for subjects with haematological diseases vs. 0.688 ± 0.156 ng/μL for healthy subjects [*P* < 0.001]) in the subjects with haematological diseases. Regarding the other GM3 species, no differences in GM3(d18:1-20:0), GM3(d18:1-22:0), GM3(d18:1-23:0), or GM3(d18:1-24:0) were found between the groups.

**Table 1:** Patient characteristics (*N* = 52)

Diagnosis	No. of patients (M/F)	Age, years (mean ± SD)	WBC (×10^9^/L)	RBC (×10^12^/L)	PLT (×10^9^/L)	monocytes (×10^9^/L)	TC (mmol/L)	CRP (mg/L)	No. of type 2 diabete mellitus (M/F)	sIL-2R (×10^3^U/mL)
**MPN**										
PV	4 (4/0)	61.8 ± 14.2	9.98 ± 6.58	5.22 ± 0.99	282 ± 128	0.27 ± 0.13	3.94 ± 1.30	0.23 ± 0.15	0	
ET	8 (3/5)	64.6 ± 15.4	10.9 ± 2.99	4.94 ± 1.15	764 ± 226	0.51 ± 0.21	4.53 ± 0.50	0.44 ± 0.35	0	
CML-CP	1 (1/0)	86.0	25.4	5.78	675	1.27	4.55	0.40	0	
**MDS**										
MDS-SLD	1 (1/0)	62.0	2.70	3.02	85.0	0.26	3.47	17.50	0	
MDS-EB-2	1 (0/1)	63.0	1.50	3.52	38.0	0.08	5.02	0.3	3(2/1)	
**AML**	2 (2/0)	46.5 ± 40.3	1.30 ± 0.42	2.82 ± 0.57	67.5 ± 62.9	0.17 ± 0.19	2.40 ± 0.15	15.8 ± 20.3	0	
**B-ALL**	2 (0/2)	50.5 ± 7.8	15.5 ± 16.4	4.25 ± 1.43	8.7 ± 8.9	0.22 ± 0.28	4.60 ± 0.59	33.4 ± 26.0	0	
**Lymphoid neoplasms**										
CLL	7 (3/4)	71.7 ± 6.1	72.9 ± 103.5	3.97 ± 0.65	175 ± 71.9	1.07 ± 0.87	4.83 ± 1.30	8.93 ± 9.41	0	2.68 ± 3.94
LPL	1 (1/0)	66.0	1.70	2.19	44.0	0.16	6.23	2.0	0	0.85
plasma cell myeloma	2 (1/1)	60.5 ± 2.1	3.70 ± 1.84	2.69 ± 0.30	186 ± 26.9	0.27 ± 0.19	4.81 ± 0.48	7.25 ± 10.1	1(0/1)	
follicular lymphoma	4 (3/1)	67.8 ± 11.3	5.70 ± 1.37	4.19 ± 0.47	315 ± 98.3	0.38 ± 0.16	4.78 ± 1.00	6.45 ± 9.03	0	0.67 ± 0.095
Mantle cell lymphoma	1 (1/0)	59.0	5.80	4.16	270	0.30	4.09	22.0	0	6.67
DLBCL	10 (6/4)	65.7 ± 10.4	7.53 ± 7.22	4.15 ± 0.42	201.9 ± 84.5	0.48 ± 0.24	5.18 ± 1.37	5.61 ± 8.25	0	1.60 ± 1.12
PTCL	1 (1/0)	82.0	11.0	3.29	163	0.28	2.30	4.10	1(1/0)	3.94
**ITP**	6 (2/4)	73.7 ± 10.7	4.0 ± 1.83	3.53 ± 0.90	26.7 ± 25.9	0.22 ± 0.16	4.81 ± 1.66	1.72 ± 1.68	0	
**PRCA**	1 (0/1)	79.0	3.6	0.89	270	0.23	4.29	2.20	1(1/0)	
**Healthy subjects**	24(10/14)	62.4 ± 10.6								

MPN, myeloproliferative neoplasms; PV, polycythemia vera; ET, essential thrombocythemia; CML-CP, chronic myeloid leukemia chronic phase; MDS-SLD, myelodysplastic syndrome with single lineage dysplasia; MDS-EB-2, MDS with excess blasts-2; AML, acute myeloid leukemia; ALL, acute lymphocytic leukemia; CLL, chronic lymphocytic leukemia; LPL, lymphoplasmacytic lymphoma; DLBCL, diffuse large B-cell lymphoma; PTCL, peripheral T-cell lymphoma, not otherwise specified; ITP, immune thrombocytopenia; PRCA, pure red cell aplasia

**Figure 1:**
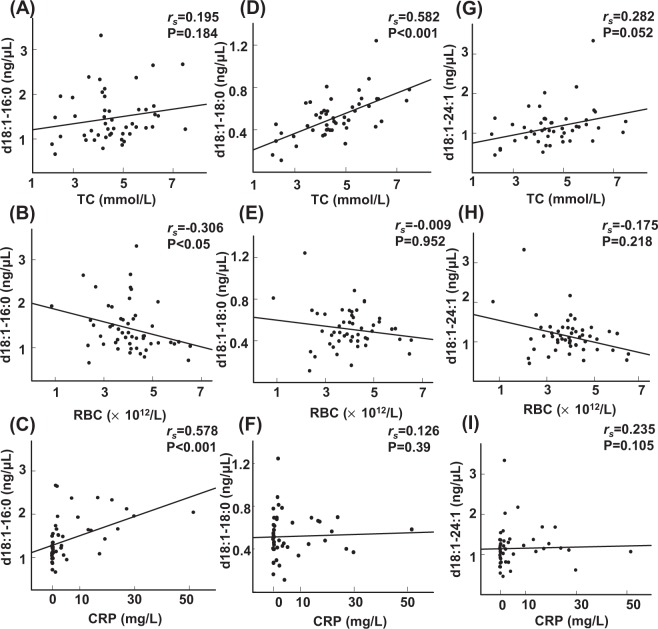
Associations between blood test values and GM3 (d18:1-16:0, d18:1-18:0, and d18:1-24:1). Correlations between GM3(d18:1-16:0) and TC (**A**), RBC (**B**), and CRP (**C**); between GM3(d18:1-18:0) and TC (**D**), RBC(**E**), and CRP (**F**); and between GM3(d18:1-24:1) and TC (**G**), RBC (**H**), and CRP(**I**) were observed. Spearman’s rank correlation was used to assess the correlations between the GM3 molecular species and the blood test values. TC, total cholesterol; RBC, erythrocytes; CRP, C-reactive protein.

Next, we investigated the correlations between the serum molecular species and various blood test values. We found several possible differences in the correlations with blood test values among the GM3 molecular species. For example, the correlations between the serum GM3 levels and the total cholesterol (TC) or C-reactive protein (CRP) levels differed among the GM3 molecular species (Fig. 1 and Table 2): the GM3(d18:1-18:0) levels were strongly correlated with the TC levels (Fig. 1D), while the GM3(d18:1-16:0) and GM3(d18:1-24:1) levels were weakly correlated with the TC levels (Fig. 1A,G). For CRP, only the GM3(d18:1-16:0) level showed a significant and moderate correlation with the CRP level (Fig. 1C). Regarding the blood cell counts, we found only GM3(d18:1-16:0) were significantly inversely correlated with the erythrocytes (RBC) count (Fig. 1B).

**Table 2:** Correlation between blood test values and serum GM3 molecular species in all measured specimens.

	WBC (×10^9^/L)	RBC (×10^12^/L)	PLT (×10^9^/L)	monocytes (×10^9^/L)	TC (mmol/L)	CRP (mg/L)
d18:1-16:0 (ng/μL)	0.103	−0.306*	−0.27	0.158	0.195	0.578***
d18:1-18:0 (ng/μL)	0.12	−0.009	0.08	0.274	0.582***	0.126
d18:1-20:0 (ng/μL)	0.096	−0.218	0.162	0.148	0.36*	0.115
d18:1-22:0 (ng/μL)	0.109	−0.084	0.162	0.168	0.6***	0.023
d18:1-23:0 (ng/μL)	0.125	−0.057	0.097	0.197	0.579***	−0.065
d18:1-24:0 (ng/μL)	0.124	0.04	0.095	0.161	0.614***	−0.158
d18:1-24:1 (ng/μL)	0.129	−0.175	0.028	0.131	0.282	0.235
total GM3 (ng/μL)	0.185	−0.162	−0.042	0.225	0.468**	0.287

Spearman’s rank correlations were used to assess the relationships between the serum GM3 molecular species and the blood test values. *P < 0.05, ** P < 0.01, ***P < 0.001.

WBC, leukocytes; RBC, erythrocytes; PLT, platelets; TC, total cholesterol; CRP, C-reactive protein.

### Serum GM3(d18:1-16:0) and GM3(d18:1-24:1) levels were higher in patients with lymphoid neoplasms

**Figure 2:**
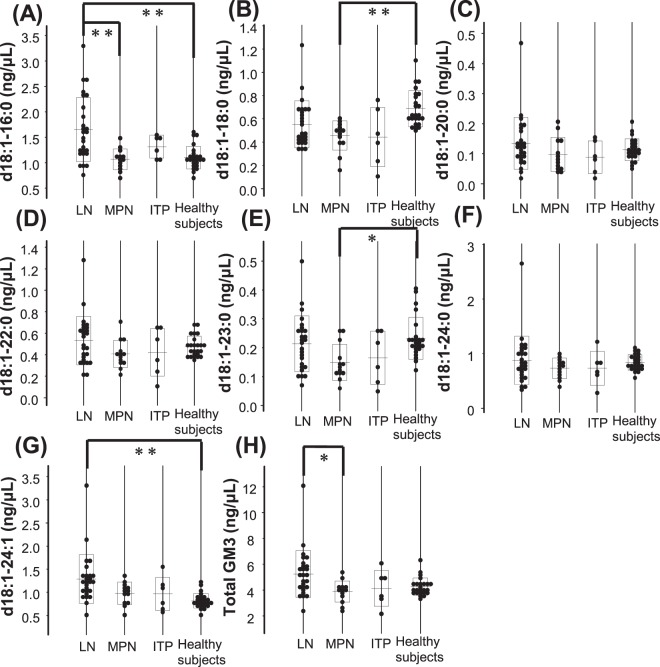
Concentrations of serum GM3 molecular species according to haematological disease groups. The serum GM3 molecular species were GM3(d18:1-16:0) (**A**), GM3(d18:1-18:0) (**B**), GM3(d18:1-20:0) (**C**), GM3(d18:1-22:0) (**D**), GM3(d18:1-23:0) (**E**), GM3(d18:1-24:0) (**F**), GM3(d18:1-24:1) (**G**), and total GM3(**H**). The haematological disease groups were LN (lymphoid neoplasms), MPN (myeloproliferative neoplasms), and immune thrombocytopenia (ITP). The Kruskal-Wallis test followed by the Games Howell test as a post-hoc test were used to compare haematological disease groups and healthy subjects. *P < 0.05, **P < 0.01.

We next compared the serum GM3 molecular species levels among the haematological disease groups. As shown in Fig. 2, although the total GM3 levels in the haematological disorder groups were not significantly different from those in the healthy subjects, some GM3 species were modulated in the subjects with haematological diseases: the GM3(d18:1-16:0) levels were higher in the lymphoid neoplasm group than in the healthy subjects and the myeloproliferative neoplasms (MPN) group, the GM3(d18:1-24:1) levels were also higher in the lymphoid neoplasm group than in the healthy subjects (Fig. 2A,G), while the GM3(d18:1-18:0) and GM3(d18:1-23:0) levels were lower in the MPN group than in the healthy subjects (Fig. 2B,E). The GM3(d18:1-24:1) levels were higher in the lymphoid neoplasm group than in the healthy subjects, even under conditions ignoring these two high expression cases.

### The correlations between the serum GM3(d18:1-16:0) or GM3(d18:1-24:1) levels and clinical parameters in patients with lymphoid neoplasms

Since the serum GM3(d18:1-16:0) and GM3(d18:1-24:1) levels were significantly higher in the patients with lymphoid neoplasms, we analyzed the potential correlations between the serum GM3(d18:1-16:0) or GM3(d18:1-24:1) levels and clinical parameters, including prognostic factors, in 26 patients with lymphoid neoplasms (Table 3). When the patients were divided into two groups according to each individual’s clinical parameters, we observed that the serum GM3(d18:1-16:0) and GM3(d18:1-24:1) levels were significantly higher in the patients with the Age Adjusted International Prognostic Index (aaIPI) of High-intermediate or High risk than in those with aaIPI of Low or Low-intermediate risk. The serum GM3(d18:1-24:1) levels were also significantly higher in the subjects with disease relapse or disease progression than in those at the time of their initial diagnosis (*P* < 0.05). Regarding the grade of lymphoma, we observed no difference between low grade lymphoma (chronic lymphocytic leukemia/lymphoplasmacytic lymphoma/plasma cell myeloma/ follicular lymphoma/ mantle cell lymphoma) and high grade lymphoma (diffuse large B-cell lymphoma/ peripheral T-cell lymphoma, not otherwise specified). These results suggest that both of the GM3 molecular species might be affected but by the degree of lymphoma infiltration and prognostic factors, not by the types of lymphoma nor age.

**Table 3:** Correlations between serum GM3 (d18:1-16:0, d18:1-24:1) levels and clinical parameters in patients with lymphoid neoplasms.

Characteristic	Patients (n)	Serum GM3 (d18:1-16:0) ng/μL	P	Serum GM3 (d18:1-24:1) ng/μL	P
**Age**					
<60 years	5	1.62 ± 0.440	0.85	1.25 ± 0.362	1.00
≥60 years	21	1.66 ± 0.678		1.30 ± 0.566	
**Disease status**					
At diagnosis	20	1.62 ± 0.653	0.53	1.17 ± 0.355	<0.05
At relapse or in regression	6	1.76 ± 0.594		1.69 ± 0.809	
**Bone marrow involvement**					
Absence	11	1.36 ± 0.346	0.06	1.16 ± 0.224	0.36
Presence	15	1.87 ± 0.713		1.38 ± 0.660	
**Extranodal involvement**					
Absence	6	1.33 ± 0.252	0.27	1.19 ± 0.183	0.93
Presence	20	1.75 ± 0.683		1.32 ± 0.594	
**B symptoms**					
Absence	21	1.64 ± 0.650	1.00	1.29 ± 0.547	0.75
Presence	5	1.69 ± 0.615		1.29 ± 0.488	
**Clinical classification**					
Low grade lymphoma	15	1.72 ± 0.752	0.838	1.30 ± 0.63	0.721
High grade lymphoma	11	1.55 ± 0.433		1.27 ± 0.37	
**Ann Arbor stage**					
I–III	6	1.34 ± 0.286	0.27	1.22 ± 0.238	1.00
IV	20	1.74 ± 0.681		1.31 ± 0.590	
**Index for lymphoma** ^**†**^					
**IPI**					
1–3	14	1.57 ± 0.569	0.88	1.39 ± 0.663	0.32
4, 5	3	1.52 ± 0.360		1.28 ± 0.0798	
**aaIPI**					
Low, Low-intermediate risk	10	1.26 ± 0.274	<0.01	1.12 ± 0.248	<0.05
High-intermediate, High risk	7	2.00 ± 0.511		1.72 ± 0.788	
**Index for CLL**					
**Rai stage**					
0–1	3	1.20 ± 0.435	0.11	0.850 ± 0.319	0.11
2–4	4	2.48 ± 0.694		1.31 ± 0.273	

Statistical significance was determined using the Mann-Whitney U test.

Low grade lymphoma includes CLL, LPL, plasma cell myeloma, follicular lymphoma, mantle cell lymphoma; high grade lymphoma includes DLBCL, PTCL.

^†^Lymphoma consists of lymphoid neoplasms excluding CLL and plasma cell myeloma.

IPI, International Prognostic Index; aaIPI, Age-Adjusted International Prognostic Index.

### Serum GM3(d18:1-16:0) levels were positively correlated with CRP or sIL-2R levels

**Figure 3:**
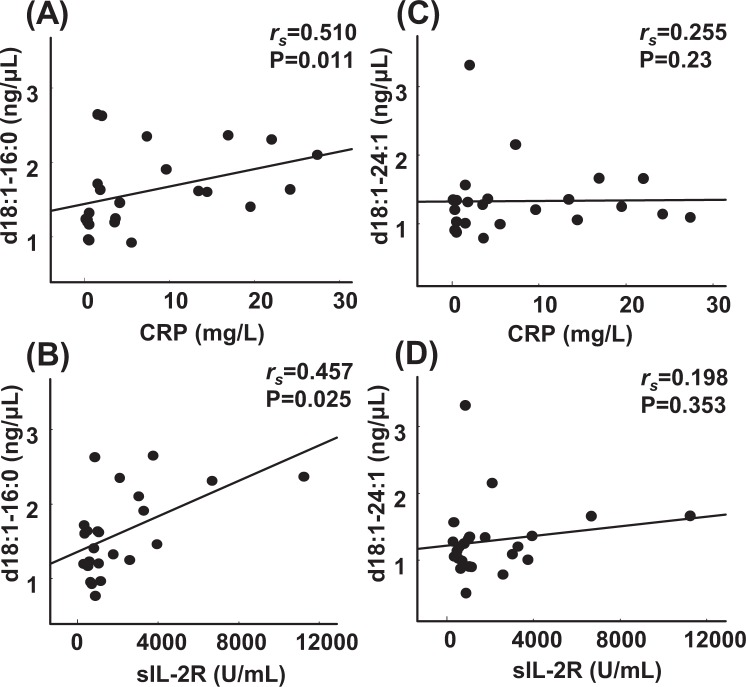
Association between blood test values related to lymphoma and GM3(d18:1-16:0 and d18:1-24:1). Correlations between GM3(d18:1-16:0) and CRP (**A**), GM3(d18:1-16:0) and sIL-2R (**B**) and between GM3(d18:1-24:1) and CRP (**C**), GM3(d18:1-24:1) and sIL-2R (**D**) were observed. Spearman’s rank correlation was used to assess the correlations between the GM3 molecular species and the blood test values related to lymphoma. CRP, C-reactive protein; sIL-2R, soluble interleukin-2 receptor.

Since the serum GM3(d18:1-16:0) and GM3(d18:1-24:1) levels were significantly higher in the patients with lymphoid neoplasms, we investigated the correlation between the serum GM3 molecular species levels and CRP, soluble interleukin-2 receptor (sIL-2R), or lactate dehydrogenase (LD), which are usually used as a biomarker for lymphoma in clinical practice. As shown in Fig. 3 and Supplementary Table S2, the GM3(d18:1-16:0) levels were significantly correlated with the levels of CRP, sIL-2R, and LD. Regarding the other GM3 molecular species, no significant correlations with any biomarkers were found.

### Principal component analysis of serum GM3 molecular species

**Figure 4:**
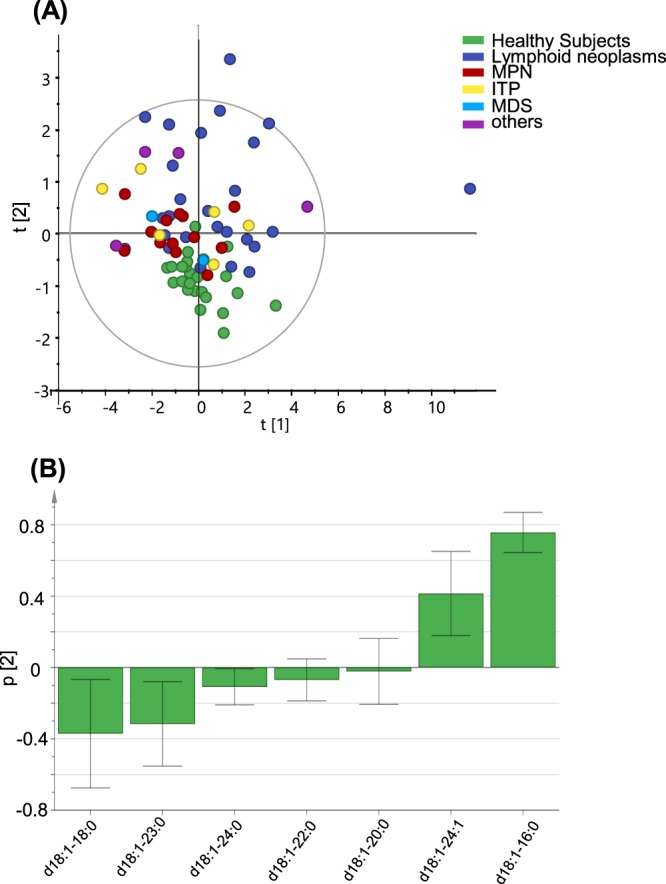
Principal component analysis of serum GM3 molecular species from subjects with haematological diseases and healthy subjects. (**A**) Scatter plot of principal component analysis scores. Subjects with lymphoid neoplasms were almost clearly separated from the healthy subjects in the t [2] axis direction. (**B**) Contributing factors to the t [2] axis in the principal component analysis. The GM3(d18:1-16:0) and GM3(d18:1-24:1) levels were significant contributing factors.

Finally, we performed a principal component analysis of serum GM3 molecular species in 52 subjects with haematological diseases and 24 healthy subjects. As shown in Fig. 4, the subjects with lymphoid neoplasms were almost clearly separated from the healthy subjects in the t[2] axis direction (Fig. 4A), for which the GM3(d18:1-16:0) and GM3(d18:1-24:1) levels were significant contributing factors (Fig. 4B).

## Discussion

GM3 is a type of GSL, which are thought to be involved in the formation of lipid rafts and therefore involved in intracellular signaling^18^. Since GM3 is known to play crucial roles in the differentiation of several blood cell lines, we investigated the association between GM3 molecular species and haematological diseases using human serum samples and LC-MS/MS. Although the present study was an observational exploratory study and we have investigated with a small number of subjects with various haematological diseases and we have not investigated the time course of GM3, especially after treatment, we obtained several interesting associations between serum levels of GM3 molecular species and haematological diseases.

First, we investigated the correlation between serum molecular species and various blood test values to investigate potential differences in the characteristics of the GM3 species and found that the correlations between the serum GM3 levels and the TC or CRP levels differed among the GM3 molecular species, and that only GM3(d18:1-16:0) were inversely correlated with the RBC count (Fig. 1 and Table 2). Regarding the correlations with TC, serum GM3 levels are reportedly affected by glucose and lipid metabolism abnormalities and visceral fat accumulation^9,13^. In our study, only the TC levels were significantly and positively correlated with all the GM3 molecular species except for GM3(d18:1-16:0) and GM3(d18:1-24:1), which agrees with the results of a previous paper^9^. Although no significant correlation between the GM3 levels and the presence of glucose intolerance or body mass index was seen in our study, these results might be explained by the study population in the present study, since none of the patients had poorly controlled hyperglycemia or obesity. Regarding the associations with the RBC count, the exacerbation of haematological diseases, which can cause anemia, might also have affected the negative correlations.

Next, we observed that the levels of GM3(d18:1-16:0) and GM3(d18:1-24:1) were significantly higher in the lymphoid neoplasm group (Fig. 2). Moreover, we found that, although the number of the subjects was small, the levels of these GM3 species were significantly higher in patients with a poor risk, as determined by the aaIPI, and the GM3(d18:1-16:0) level was significantly and positively correlated with the levels of CRP, sIL-2R, and LD (Fig. 3). This study is the first to demonstrate an association between a GM3 species and lymphoma in human subjects. Several reports have proposed that the CRP level might predict survival in patients with lymphoma, although the role of CRP as a biomarker for lymphoma has not been established^19,20^. Since we excluded patients with infections, the CRP level could be used as a possible biomarker for lymphoma, rather than as an inflammatory marker. These results suggested that both of the GM3 molecular species might be associated with prognostic factors or the degree of lymphoma infiltration.

Regarding the association between serum GM3 molecular species levels and human diseases, Veillon, L. *et al*. reported that the serum GM3(d18:1-20:0) level was significantly elevated in subjects with visceral fat accumulation accompanied with hyperglycemia and dyslipidemia among the GM3 molecular species we have measured in the present study, while GM3(d18:1-16:0) and GM3(d18:1-24:1) levels were not altered^13^. A basic study also demonstrated the possible physiological importance of GM3 molecular species; the GM3(d18:1-16:0) content increased and the GM3(d18:1-24:1) content decreased during differentiation of mouse C2C12 myoblast cells^21^. Further studies are needed to elucidate the specific roles of GM3 molecular species.

In addition to the results for lymphoma, other interesting findings included significantly lower serum GM3(d18:1-18:0) and GM3(d18:1-23:0) levels in the MPN group. Since these GM3 molecular species were not associated with the classification of diseases and blood test values, we could not discuss the underlying mechanisms at present. Further studies are needed to investigate the potential usefulness of these GM3 species as biomarkers for MPN.

Since this study was an observational exploratory study, we could not elucidate the mechanisms underlying these results. Possible confounding factors other than age and gender (e.g. types of lymphoid neoplasms) might underlie the significant relationships of the GM3 molecular species levels. Further studies involving larger numbers of subjects examined for longer periods and basic studies investigating the association between specific GM3 species and lymphoma are necessary to investigate the possible clinical usefulness of GM3(d18:1-16:0) and GM3(d18:1-24:1) as biomarkers for lymphoma.

In summary, in our exploratory study with haematological diseases, GM3 molecular species showed different distribution among disease groups, and serum GM3(d18:1-16:0) and GM3(d18:1-24:1) might be associated with lymphoma.

## Methods

### Subjects

We enrolled 66 patients with haematological diseases who visited the Department of Hematology and Oncology, the University of Tokyo Hospital (Tokyo, Japan) in 2011. We collected serum samples from the patients prior to the start of chemotherapy or from those who received supportive therapy only, have not been transfused for at least 2 weeks. Patients with infectious disorders were excluded. However, we cannot completely exclude the possible effects of blood transfusions, considering the life span of red blood cells. Therefore, in the present study, we analyzed the data of 52 patients, excluding the patients who had received transfusions. The serum samples used in this study were residual samples remaining after the completion of the requested clinical laboratory tests. In Table 3, the patients with lymphoid neoplasms were staged; among the patients with lymphoma, the clinical stage was assessed according to the Ann Arbor classification^22^, the prognostic factors were assessed according to the International Prognostic Index (IPI) and aaIPI, and the performance status was assessed using the Eastern Cooperative Oncology Group (ECOG) scale. Patients with CLL were staged according to the Rai stage^23^.As a control group, blood was collected from 24 healthy adult volunteers. All the participants provided written informed consent prior to their inclusion in the study. This study was approved by the ethics committee of The University of Tokyo (3459-2) and all experiments were performed in accordance with relevant guidelines and regulations.

### LC-MS/MS analysis

The total lipid contents were extracted from the serum samples as follows: 9.6 ng of deuterium-labeled GM3 (d18:1-[²H]16:0) was added as an internal standard to 20 μL of serum, and the solution was dissolved in 200 µL of methanol. The mixture was then mixed at 1,200 rpm at room temperature for 1 hour using a vortex machine, then centrifuged at 15,000 rpm at 4 °C for 20 min. The supernatant (100 µL) was then transferred to a glass autosampler vial.

GM3 molecular species were quantified using high-performance liquid chromatography (HPLC) coupled with electrospray ionization tandem**-**mass spectrometry (MS/MS) in multiple reaction monitoring (MRM) negative ionization mode as described in the previous paper^13^ with minor modification as follows. The triple-stage quadrupole (TSQ) Vantage AM instrument (Waltham, MA) was calibrated by directly infusing a mixture of GM3 species extracted from milk, and all ion source parameters and ionization conditions were optimized to improve sensitivity. Eight microliters of the supernatant were injected into a Thermo Fisher Accela 1250 HPLC pump (Waltham, MA) and separated using a C18 column (InertSustain C18, 3 µm 1.0 × 50 mm; GL Science, Japan). The gradient program started with 90% solvent A (20% H_2_O/50% 2-propanol/30% methanol containing 0.1% acetic acid and 0.1% ammonia) for 1 min, then ramped to 100% solvent B (52% 2-propanol/48% methanol containing 0.1% acetic acid and 0.1% ammonia) over 7 min. One hundred percent solvent B was maintained for 6 min, then the solvent was returned to 90% solvent A over 1 min and held there for 5 min. The flow rate throughout the duration of the chromatographic run was 60 μL/min. A potential of −2500 V was applied between the ion source and the electrospray needle, and nitrogen gas was used. The vaporizer temperature was 90 °C, the sheath gas pressure was 25 (arbitrary units), the ion sweep gas pressure was 0 (arbitrary units), the auxiliary gas pressure was 5 (arbitrary units), the capillary temperature was 208 °C, the declustering voltage was 0, the collision pressure was 1.5 mTorr, the S-lens RF amplitude was 310, and the collision energy was 50 eV for GM3 molecular species. A 0.05-s scan time was used, data were collected in profile mode, and the peak widths were Q1 full width at half maximum (FWHM) 1.0 and (FWHM) 1.0. The MS/MS transitions are listed in Supplementary Table S1. We calculated the concentrations of GM3 molecular species from the area ratio relative to the internal standard. The total GM3 values were calculated by the summation of the concentrations of the 7 molecular species that were detected.

### Statistical analysis

The data were statistically analyzed using SPSS (Chicago, IL) or SIMCA (MKS Umetrics). The results are expressed as the mean ± SD. We performed nonparametric analyses because the equality of the variance was rejected by the Shapiro-Wilk test for some of the parameters or analyses; a comparison between two groups was performed using the Mann-Whitney U test and the Pearson’s chi-square test, and correlations were determined using the Spearman correlation test. The values obtained from three groups were compared using the Kruskal-Wallis test followed by the Games Howell test as a post-hoc test. A value of *P* < 0.05 was regarded as denoting statistical significance in all the analyses. The principal components analysis (PCA) was statistically analyzed using SIMCA (MKS Umetrics).

## Supplementary information

Supplemental Table S1,2

## Data Availability

The datasets generated or analyzed during the current study are available on reasonable request.
