# Supplementary material for: Serum GM3(d18:1-16:0) and GM3(d18:1-24:1) levels may be associated with lymphoma: An exploratory study with haematological diseases
Source: Sci Rep. 2019 Apr 19;9:6308. doi: 10.1038/s41598-019-42817-3 (PMC6474875; doi:10.1038/s41598-019-42817-3)
Supplement: Supplementary file 1 — Supplemental Table S1,2 [file 41598_2019_42817_MOESM1_ESM.pdf]

<u>SUPPLEMENTAL INFORMATION:</u>

Serum GM3(d18:1-16:0) and GM3(d18:1-24:1) levels may be associated with lymphoma: An exploratory study with haematological diseases.

Masako Nishikawa[1], Makoto Kurano[1], Takahiro Nitta[2], Hirotaka Kanoh[2], Jin-ichi Inokuchi[2], and Yutaka Yatomi[1]

1 Department of Clinical Laboratory Medicine, Graduate School of Medicine, The University of Tokyo, Tokyo 113-8655, Japan;

2 Division of Glycopathology, Institute of Molecular Biomembrane and Glycobiology, Tohoku Medical and Pharmaceutical University, Sendai, Miyagi 981-8558, Japan.

Supplemental Table S1. Analysis conditions for LC-MS/MS

	Molecular Species	Q1 m/z	Q3 m/z	CE(eV)	S-lens RF amplitude
GM3					
$[M-H]^-$	d18:1-16:1	1149.7	289.9	50	310
	d18:1-16:0	1151.7	289.9	50	310
	d18:1-h16:1	1165.7	289.9	50	310
	d18:1-h16:0	1167.8	289.9	50	310
	d18:1-18:1	1177.7	289.9	50	310
	d18:1-18:0	1179.7	289.9	50	310
	d18:1-[^{2}H]16:0	1182.9	289.9	50	310
	d18:1-h18:1	1193.8	289.9	50	310
	d18:1-h18:0	1195.7	289.9	50	310
	d18:1-20:1	1205.8	289.9	50	310
	d18:1-20:0	1207.8	289.9	50	310
	d18:1-21:1	1219.8	289.9	50	310
	d18:1-21:0	1221.8	289.9	50	310
	d18:1-h20:0	1223.8	289.9	50	310
	d18:1-22:1	1233.8	289.9	50	310
	d18:1-22:0	1235.8	289.9	50	310
	d18:1-h21:0	1237.8	289.9	50	310
	d18:1-23:1	1247.8	289.9	50	310
	d18:1-23:0	1249.8	289.9	50	310
	d18:1-h22:0	1251.8	289.9	50	310
	d18:1-24:1	1261.8	289.9	50	310
	d18:1-24:0	1263.8	289.9	50	310
	d18:1-h23:0	1265.8	289.9	50	310
	d18:1-25:1	1275.8	289.9	50	310
	d18:1-h24:1	1277.8	289.9	50	310
	d18:1-h24:0	1279.8	289.9	50	310
	d18:1-26:1	1289.8	289.9	50	310
	d18:1-26:0	1291.8	289.9	50	310
	d18:1-h25:0	1293.8	289.9	50	310
	d18:1-h26:1	1305.8	289.9	50	310
	d18:1-h26:0	1307.9	289.9	50	310

Q, quadrupole; CE, collision energy; S-lens, stacked-ring ion guide; RF, radio frequency.

Supplemental Table S2. Correlation between serum GM3 molecular species and blood test values in patients with lymphoid neoplasms

	WBC ($\times 10^9$/L)	RBC ($\times 10^{12}$/L)	PLT ($\times 10^9$/L)	monocyte ($\times 10^9$/L)	TC (mmol/L)	CRP (mg/L)	LD (U/L)	sIL-2R (U/mL)
d18:1-16:0 (ng/μL)	0.27	-0.415	-0.372	0.174	0.057	0.51*	0.533**	0.457*
d18:1-18:0 (ng/μL)	-0.008	-0.253	0.076	0.148	0.643***	0.054	-0.016	-0.274
d18:1-20:0 (ng/μL)	0.066	-0.251	0.027	0.078	0.272	0.09	-0.067	-0.176
d18:1-22:0 (ng/μL)	0.132	-0.078	0.027	0.097	0.554**	-0.006	-0.005	-0.184
d18:1-23:0 (ng/μL)	0.084	0.019	-0.035	0.057	0.566**	-0.333	0.066	-0.381
d18:1-24:0 (ng/μL)	0.03	-0.057	-0.121	-0.004	0.563**	-0.322	-0.116	-0.251
d18:1-24:1 (ng/μL)	0.02	-0.495	-0.249	-0.063	0.042	0.255	0.14	0.198
total GM3 (ng/μL)	0.24	-0.257	-0.236	0.144	0.372	0.203	0.345	0.109

Spearman's rank correlations were used to assess the relationships between the serum GM3 molecular species and the blood test values in patients with lymphoid neoplasms. * $P < 0.05$, ** $P < 0.01$, *** $P < 0.001$.

WBC, leukocytes; RBC, erythrocytes; PLT, platelets; TC, total cholesterol; CRP, C-reactive protein; LD, lactate dehydrogenase; sIL-2R, soluble interleukin-2 receptor.
